# Duodenal Ulceration Erosion Into the Common Bile Duct Status Post-Roux-en-Y Gastric Bypass: A Case Report

**DOI:** 10.7759/cureus.62496

**Published:** 2024-06-16

**Authors:** Andrew Harris, Nathan Chance, Ian Fillerup, Eric Morrison, Andrea Goethals

**Affiliations:** 1 Internal Medicine, Oklahoma State University Medical Center, Tulsa, USA; 2 Obstetrics and Gynecology, McLaren Greater Lansing Hospital, Lansing, USA; 3 Internal Medicine, Baylor Scott & White Medical Center, Temple, USA; 4 General Surgery, Ascension Genesys Hospital, Grand Blanc, USA

**Keywords:** perforated peptic ulcer, common bile duct (cbd), gastroduodenal artery, peptic ulcer disease, roux-en-y gastric bypass

## Abstract

Peptic ulcer disease (PUD) affects approximately four million people worldwide. The most common etiologies of PUD are *Helicobacter pylori* (H. pylori) infections, chronic nonsteroidal anti-inflammatory drug (NSAID) use, and smoking. A rare cause of ulcer formation is documented in patients following Roux-en-Y gastric bypass (RYGB) procedures. Delayed treatment of these ulcers can further lead to ulcer perforation, erosion of the gastroduodenal artery (GDA), and fistula formation between the biliary structures and the gastrointestinal tract. Herein, we discuss the case of a 69-year-old female with an ulcer perforation 19 years after RYGB, resulting in an atypical ulcer erosion of the common bile duct without fistula formation.

## Introduction

Roux-en-Y gastric bypass (RYGB) surgery is a metabolic surgery used to treat obese individuals who struggle with weight loss. According to the National Institute of Health, an RYGB surgery is indicated for each of the following situations: individuals with a body mass index (BMI) >40 kg/m^2^ regardless of comorbidities, a BMI >35 kg/m^2^ with at least one or more comorbidities, and lastly, a BMI >30 kg/m^2^ with type II diabetes that is not well controlled with medications or lifestyle changes [1]. The general overview of the procedure includes reducing the stomach into a small gastric pouch, followed by anastomosing the pouch to the jejunum and jejuno-jejunostomy, thus bypassing the remnant stomach, duodenum, and proximal jejunum [2]. Circumventing the antrum may reduce the secretion of gastrin, therefore increasing complications that arise after RYGB surgery, including bleeding, gallstones, dumping syndrome, nutrient deficiency, herniation, fistula formation, and ulcer formation [3,4]. The development of a duodenal ulcer after RYGB surgery is a complication that can eventually lead to bleeding or perforation if not treated promptly.

Peptic ulcer disease (PUD) is the most common etiology of a duodenal ulcer but has decreased recently due to the eradication of *Helicobacter pylori *(*H. pylori*) and the use of proton-pump inhibitors [5]. PUD affects approximately four million people worldwide, with 5% of those individuals experiencing ulcer perforation [5]. Laverty et al. recently reported that 73% of the peptic ulcers formed in patients with RYGB surgeries involved the duodenum [6]. Perforation of a duodenal ulcer will present with symptoms more alarming than generalized abdominal pain, such as melena, hematemesis, and anemia. Perforation of an ulcer can become further complicated by eroding into nearby structures, most commonly involving the gastroduodenal artery (GDA) [7]. Herein, we describe the rare finding and a novel surgical approach of a patient who presented with a duodenal ulcer perforation after RYGB with erosion into the common bile duct (CBD).

## Case presentation

A 69-year-old female patient with a history of Class II obesity (BMI 35.3 kg/m^2^) and an RYGB 19 years ago, resulting in a weight loss of 60 lb, presented to the Emergency Department with achy, epigastric pain radiating to the back, rated as a 6/10 on the pain scale. The patient reported multiple episodes of melena and dark, pasty stools over the past week. Additionally, she mentioned having weakness, dizziness, and near syncope. The outpatient gastroenterology evaluation for her anemia included an esophagogastroduodenoscopy (EGD) and a colonoscopy prior to admission. The evaluation with EGD was negative, while the colonoscopy showed a superficial ulcer in the colon without evidence of bleeding. On presentation, the patient was hemodynamically stable with no skin pallor. The abdomen was soft, minimally tender, and non-distended, with normoactive bowel sounds. Her complete blood count on admission showed decreased hemoglobin, hematocrit, and mean corpuscular hemoglobin levels (Table 1).

**Table 1:** Laboratory findings on admission. INR: international normalized ratio; PG: picogram.

Lab	Result	Reference
Serum sodium	133	136–144 mmol/L
Serum glucose	203	70–99 mg/dL
Serum calcium	8.3	8.4–10.2 mg/dL
Serum albumin	3.0	3.5–5.0 g/dL
Serum blood urea nitrogen	44	8–26 mg/dL
Serum creatinine	1.85	0.44–1.00 mg/dL
Estimated glomerular filtration rate	27.03	60.00–130 mL/min
Lipase	45	22–51 U/L
Hemoglobin	5.6	11.0–16.2 g/dL
Hematocrit	16.7	36.0–46.0 %
Mean corpuscular volume	74.8	80.00–100.0 fL
Mean corpuscular hemoglobin	25.0	29.0–34.0 PG
Protime	16.5	10.1–12.5 sec
INR	1.49	0.80–1.20

**Figure 1:**
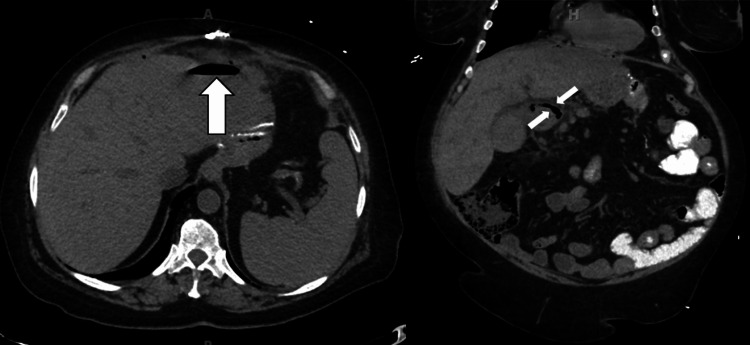
Perforated viscus with anterior lucency indicative of an air-fluid level in a subhepatic fluid collection with adjacent small foci of intraperitoneal free-air along the anterior abdomen.

A computed tomography (CT) scan of the abdomen and pelvis without intravenous contrast revealed an 8.8 cm fluid pocket with air-fluid levels in the epigastric/subdiaphragmatic area consistent with a perforated viscus and abscess (Figure 1).

**Figure 2:**
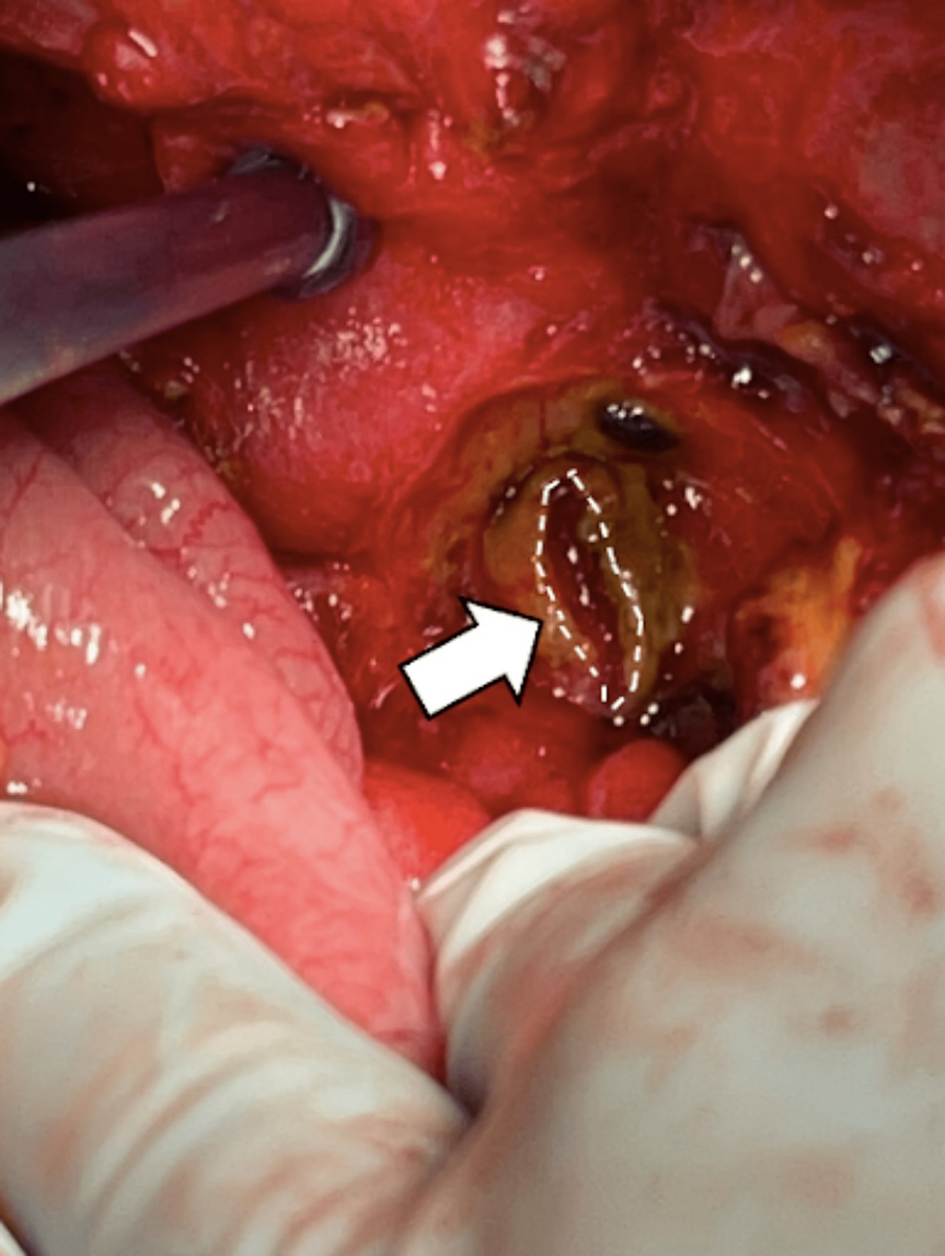
With retraction of the small bowel along an anterior plane, visualization of the posterior ulcer during laparotomy is evident with the common bile duct involved.

These findings warranted an exploratory laparotomy. The exploration of the abdomen revealed extensive adhesions from prior surgery with bile leakage from a perforated ulcer in the posterior aspect of the proximal duodenum with no signs of active bleeding from the ulcer bed. The subhepatic abscess was removed, and adhesiolysis was performed. At the base of the ulcer perforation, there was significant erosion into the CBD with longitudinal erosion of the duct and free-flowing bile (Figure 2).

Open cholecystectomy was performed with no signs of leakage from the cystic artery or cystic duct. No bleeding was noted intraoperatively from the GDA or ulcer bed. Due to excessive inflammation and poor visualization of the GDA, ligation of the vessel was not feasible. A 12-French biliary T-tube was placed in the CBD opening and secured with interrupted polydioxanone suture to close the bile duct over the T-tube and allow for external drainage of the biliary tree. Resection of the duodenal ulcer was not considered because of its involvement with the bile duct and close approximation to the ampulla along the medial surface. For decompression and drainage of pancreatic and gastroduodenal secretions, a 26-French Malecot catheter was placed into the gastric remnant and advanced across the pylorus with its tip positioned distal to the duodenal ulcer. A 20-French round Blake channel drain (Ethicon, Bridgewater, New Jersey) was placed in the right upper abdomen and then into the foramen of Winslow for dependent drainage. Interrupted silk sutures were used to approximate the portion of the duodenal wall that was disrupted by the ulcer. Due to inflammatory changes in this area, this was further reinforced by placing an omental patch over the ulcer site. Lastly, a 10-flat Jackson-Pratt drain (Cardinal Health, Dublin, Ohio) was laid anterior to the gastric remnant in the subhepatic space where the previous abscess was noted and brought out through a small incision on the left upper abdomen. The biliary T-tube was externalized through a small incision in the right upper quadrant as well. The midline abdomen was then closed with a running suture.

**Figure 3:**
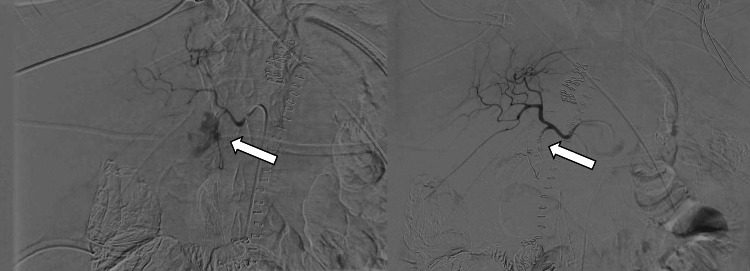
Angiogram showing perforation of the gastroduodenal artery with active contrast extravasation on the left. Post-coil embolization of the gastroduodenal artery demonstrated no residual bleeding on the right.

On postoperative day one, the patient developed hemodynamic instability. Hemorrhagic shock was suspected with the persistence of anemia and frank blood oozing from the gastroduodenostomy Malecot and Blake drains. A CT angiogram was performed and revealed an active hemorrhage from the GDA. At that time, a massive transfusion protocol was then initiated, interventional radiology was consulted, and angioembolization of the GDA was performed (Figure 3).

## Discussion

According to Plitzko et al.'s review of the literature, 54 patients were reported to have perforated duodenal ulcers in the excluded segments following RYGB, and the incidence is not known [8]. Commonly reported symptoms by patients include right upper quadrant pain, melena, and hematemesis, with a sudden onset of abdominal pain [8]. PUD perforations after RYGB most commonly involve the GDA [7]. While the prevalence of ulcer formation in post-RYGB patients is a rare finding, it should promptly be considered in patients post-RYGB who are presenting with right upper quadrant pain and signs of hemodynamic instability. The first diagnostic step in the investigation of perforated peptic ulcer is an upright abdominal radiograph, with a positive finding being the discovery of air beneath the diaphragm, also known as a pneumoperitoneum [7]. Although very specific for a perforation, this finding on imaging has poor sensitivity and is an extremely variable finding [7]. For this reason, abdominal CT is the preferred method for diagnosis [7]. Laparoscopy and direct visualization of the pathology are the most definitive methods for diagnosing a perforated ulcer [7]. To our knowledge, our case report is the first to discuss a duodenal ulcer perforation after an RYGB that extends into the CBD, with subsequent documentation of a novel surgical approach for its management.

Although the etiology of peptic ulcers is diverse, a proposed mechanism for the underlying pathogenesis is an imbalance between defensive (mucus-bicarbonate layer) and aggressive factors (hydrochloric acid) that provide the optimal environment for alimentary digestion. The most common causes of peptic ulcers include infection from *H. pylori*, smoking, and nonsteroidal anti-inflammatory drugs [9]. Additionally, for post-RYGB patients, hypotheses regarding physiological changes have also been suggested to increase the risk of developing an ulcer. Bjorkman et al. proposed that a gastrectomy can lead to lower gastrin levels, ultimately increasing acid production [3].

Without appropriate treatment, these ulcers may compromise the integrity of the bowel wall, leading to bowel perforation, with the most common complication of perforated PUD being hemorrhage [7]. Untreated hemorrhage can manifest as hematemesis or melena, leading to anemia in patients with chronic PUD. Subsequently, unresolved management of bleeding is associated with 40% of ulcer-related deaths [7]. Another complication associated with chronic ulceration is the formation of a fistula between the small intestine and the biliary tract. Although these are less common sequelae, numerous variations of fistula formation exist, namely cholecystoduodenal, choledochoduodenal, cholecystocolonic, and cholecystogastric [10]. However, the lack of fistula formation in the setting of a duodenal ulcer with the involvement of the CBD is an undocumented occurrence.

Currently, there is no conclusive evidence as to whether gastrectomy with duodenal resection, oversewing, or Graham patches are the gold standard approaches to repairing complicated perforated peptic ulcers [7,8]. According to Plitzko et al.'s analysis, 48% of 50 cases underwent a remnant gastrectomy with resection of the first portion of the duodenum; meanwhile, 37% of the 50 cases underwent either oversewing or Graham patch procedures for treatment [8]. While remnant gastrectomy would be a definitive solution for future ulcers, data on these procedures are limited. Furthermore, PUD perforation post-RYGB with CBD erosion is a rare complication, and there are currently no recommendations or guidelines for appropriate management.

Limitations and improvements

In our approach, one area of concern is the placement of the Malecot drain. It could be ulcerogenic as it crosses the surface of the ulcer within the lumen. Additionally, had a visualization of the GDA been adequate, we would have performed prophylactic ligation of the artery to prevent blood loss. However, the GDA was not visualized or ligated at the initial operation due to the extensive adhesions and inflammation associated with the ulcer and the adjacent abscess cavity. During the immediate postoperative period, the patient became hemodynamically unstable, which required activation of a massive transfusion protocol and angioembolization of the GDA for adequate hemostasis. Optimal preemptive treatment would have been to ligate the GDA before closure or prophylactically embolize the GDA immediately post-op. Lastly, our approach to resolving this complicated ulceration has yet to be validated by reproduction through other surgeons.

## Conclusions

Currently, there is no gold standard for treating or managing ulcer perforation that results in contiguous erosion of the GDA and CBD post-RYGB without evidence of fistula formation. Ulcer perforation should be highly suspected in patients with the acute onset of upper quadrant abdominal pain, hematemesis, melena, and fatigue post-RYGB. Our case demonstrated the progressive complications of ulcer perforation, in addition to an undocumented finding and surgical approach that have not been discussed in the literature to our knowledge.
